# Tissue-Specific Distribution of Legacy and Emerging Organophosphorus Flame Retardants and Plasticizers in Frogs

**DOI:** 10.3390/toxics9060124

**Published:** 2021-05-31

**Authors:** Yin-E Liu, Xiao-Jun Luo, Ke-Lan Guan, Chen-Chen Huang, Xue-Meng Qi, Yan-Hong Zeng, Bi-Xian Mai

**Affiliations:** 1 State Key Laboratory of Organic Geochemistry and Guangdong Key Laboratory of Environmental Resources Utilization and Protection, Guangzhou Institute of Geochemistry, Chinese Academy of Sciences, Guangzhou 510640, China; liuyine@gig.ac.cn (Y.-E.L.); guankl@gig.ac.cn (K.-L.G.); Huangchenchen@gig.ac.cn (C.-C.H.); qixuemeng@gig.ac.cn (X.-M.Q.); Zengyh@gig.ac.cn (Y.-H.Z.); nancymai@gig.ac.cn (B.-X.M.); 2 University of Chinese Academy of Sciences, Beijing 100049, China; 3 Guangdong-Hong Kong-Macao Joint Laboratory for Environmental Pollution and Control, Guangzhou Institute of Geochemistry, Chinese Academy of Sciences, Guangzhou 510640, China; 4 CAS Center for Excellence in Deep Earth Science, Guangzhou 510640, China

**Keywords:** organophosphorus flame retardants, plasticizers, tissue-specific accumulation, frog

## Abstract

Five types of tissues, including the liver, kidney, intestine, lung, and heart, were collected from black-spotted frogs and bullfrogs to study the tissue-specific accumulation of organophosphorus flame retardants (PFRs) and plasticizers. Thirteen PFRs and nine plasticizers were detected, with average total concentrations of 1.4–13 ng/g ww and 858–5503 ng/g ww in black-spotted frogs, 3.6–46 ng/g ww and 355–3504 ng/g ww in bullfrogs. Significant differences in pollutant concentrations among different tissues in the two frog species were found, indicating the specific selectivity distribution of PFRs and plasticizers. Overall, liver tissues exhibited significantly higher pollutant concentrations. The pollutant concentration ratios of other tissue to the sum of liver tissue and other tissues (OLR, *C*_other_/(*C*_other_ + *C*_liver_)) corresponding to male frogs were significantly greater than those of females, suggesting that male frogs could have higher metabolic potentials for PFRs and plasticizers. No obvious correlations between OLR and log *K*_OW_ were found, indicating that the other factors (e.g., bioaccumulation pathway and metabolism) besides lipophicity could influence the deposition of PFRs and plasticizers in frog livers. Different parental transfer patterns for PFRs and plasticizers were observed in frogs when using different tissues as parental tissues. Moreover, the liver tissues had similar parental transfer mechanism with muscle tissues.

## 1. Introduction

Organophosphorus flame retardants (PFRs), known as a suitable replacement of the banned brominated flame retardants, have been extensively applied to a wide variety of consumer and industrial products in the last decade, acting as flame retardants and plasticizers, as well as antifoaming agents [1,2]. Plasticizers, including the legacy phthalates and novel alternative plasticizers, are primarily used in various application, such as polyvinyl chloride, food containers, and electronics products [3]. PFRs and plasticizers are semi-volatile organic compounds and are commonly applied as non-chemically bounded end-additive in materials, and are thus, prone to escape from the products and release into the environment [1,3]. Consequently, they have been ubiquitously found in different environmental compartments at considerable levels, such as water [4,5,6,7,8], soil and sediment [9,10,11,12], air and dust [13,14,15,16,17,18,19], as well as the biota [20,21,22,23,24,25,26]. PFRs and plasticizers are the current environmental concerns of many researchers due to their non-negligible residual levels in environments, bioaccumulation characteristics, and biological toxicities [1,3].

Chemical pollution has been considered as a crucial cause for the decrease in global numbers and the increase in morphological abnormalities of amphibians [27,28,29]. Frogs are important amphibian vertebrates, and are often regarded as a meaningful environmental indicator organism owing to their unique environmental sensitivity [29]. Nevertheless, the number of vertebrate ecotoxicology studies using amphibious frogs as experimental subjects is far less than that of other vertebrates (i.e., fish, mice) [29]. As known to the authors, most of existing studies about the occurrence and fate of PFRs and plasticizers were mainly devoted to the aquatic biota, like fish, where the muscle tissue was commonly used as the target tissue.

In the last few years, some laboratory studies have reported the tissue-specific bioconcentration and distribution of PFRs in vertebrate fish [25,30,31]. A few field studies have also investigated the tissue-specific bioaccumulation potential of PFRs and plasticizers in fish [20,21,26,32,33]. However, the current information about the accumulation potential of PFRs and plasticizers in amphibian frog is scarce. Only our recent research has found significant species- and sex-differences in the accumulation of PFRs and plasticizers in frogs, by investigating the concentrations and composition patterns of pollutants in muscle and egg/gonad tissues [24]. In this study, to fill in the gaps and provide a comprehensive understanding on internal exposure of PFRs and plasticizers in amphibian frogs, thirteen PFRs and nine plasticizers were determined in five other tissues (including liver, kidney, intestine, lung, and heart) of black-spotted frogs and bullfrogs to investigate the tissue-specific distribution and accumulation patterns of these contaminants.

## 2. Materials and Methods

### 2.1. Sample Collection

In April 2019, 25 black spotted frogs (*Rana nigromaculata*, 11 females and 14 males) and 10 bullfrogs (*Rana catesbeiana*, 5 females and 5 males) were collected at an e-waste contaminated site in Longtang Town, Qingyuan County, Guangdong, South China. Detailed information about these two frog species, such as body weight and length, has been shown in our previous study [24]. Different tissues, including liver, kidney, intestine, lung, and heart, were carefully dissected from each frog. Tissue samples from each bullfrog were analyzed separately. Each type of tissues from female and male black-spotted frogs was pooled into three composite samples. A total of 80 tissue samples were analyzed in this study. The specific number of samples is shown in Table 1 and Table 2. All studied samples were kept at −20 °C until analysis. 

### 2.2. Chemical Analyses

The PFR and plasticizer analysis procedures were performed as previously reported [24,34]. In brief, about 100 mg of dry tissue sample (ISs) was ultrasonically extracted twice with 2.5 mL of acetonitrile/toluene (*v*/*v*, 9/1) after spiking with surrogate standards. Clean-up was achieved by solid-phase extraction using a Florisil^®^ ENVI cartridge (500 mg, 3 mL), which was conditioned with acetone (ACE), ethyl acetate (EtAC), and hexane. After loading the extract, the cartridge was washed with 12 mL of dichloromethane/hexane (*v*/*v*, 1/4), and then eluted with 10 mL of EtAC and 8 mL of ACE. Finally, the eluate was evaporated to dryness and replaced with methanol, and spiked with triamyl phosphate for LC-MS/MS analysis. In addition, 20 µL of this final mixture was transferred and mixed with 80 µL of EtAC for GC-MS analysis. Detail information about the target analytes and surrogate standards and instrument analyses were presented in Section S1 and Table S1 of the Supporting Information (SI).

### 2.3. Quality Assurance/Quality Control (QA/QC)

The recoveries of native standards in triplicates of spiking samples were ranged from 66% to 126%. The relative standard deviations of the analytes in three replicates were all less than 15%. One procedural blank sample was tested in parallel for every fifteen samples in the process of samples treatment. The averages of blank contamination were 0.025–1.8 ng/g ww for PFRs, and 0.19–121 ng/g ww for plasticizers. The blank values were subtracted from the detected results. Limits of quantitation (LOQs) of PFRs and plasticizers were 0.032–2.5 ng/g ww, and 0.29–266 ng/g ww, respectively. In addition, the recoveries of ISs in the analyzed samples were 70–111%. Detailed data on procedural blank levels and LOQs of each targeted chemical, as well as recoveries of each IS in the analyzed samples are listed in Tables S2 and S3 of the SI.

### 2.4. Statistical Analysis

Statistical analyses were performed using IMB SPSS Statics 19.0 and Origin 8.5 software. Independent samples t-tests were used to compare concentrations and compositions between two groups. One-way analysis of variance (ANOVA) was used to compare the contaminant patterns among different tissues. Pearson’s correlation analyses were conducted to explore the relationships on pollutant concentrations among different tissues, between pollutant concentrations and physiological parameters of bullfrogs, and parental transfer potential and log *K*_OW_ values. Significance were considered as *p* < 0.05.

## 3. Results and Discussion

### 3.1. Occurrences of PFRs and Plasticizers in Different Frog Tissues

Detailed concentrations of PFR and plasticizer analytes in the five investigated tissues of black-spotted frogs and bullfrogs are presented in Table 1 and Table 2. The average concentrations of total PFRs varied from 1.4 ng/g ww (for the male lungs) to 13 ng/g ww (for the female livers) in black-spotted frogs, and from 3.6 ng/g ww (for the male lungs) to 46 ng/g ww (for the female livers) in bullfrogs. The total average plasticizer concentrations ranged from 858 ng/g ww (for the male hearts) to 5503 ng/g ww (for the female livers) in black-spotted frogs, and from 355 ng/g ww (for the female lungs) to 3504 ng/g ww (for the female livers) in bullfrogs. To obtain a comprehensive understanding of the tissue distribution of PFRs and plasticizers in frogs, we analyzed these five investigated tissues combined with the muscle and egg/gonad tissues, as reported in our previous study [24], in the following discussion section.

**Figure 1:**
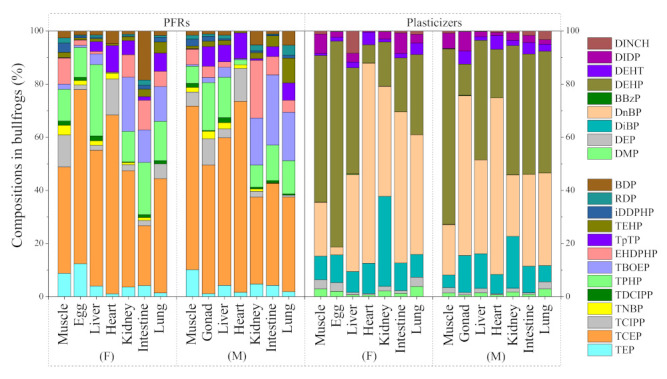
Compositions of PFRs and plasticizers in each tissue of black-spotted frogs and bullfrogs. F and M represent female and male, respectively. Data on the tissues of muscle and egg/gonad were taken from our previous study [24].

The total concentrations and compositional profiles of PFRs and plasticizers in each tissue of black-spotted frogs and bullfrogs are presented in Figure S1 and Figure 1, respectively. Significant differences in concentrations of both ∑PFRs and ∑plasticizers were found among seven different tissues, whether for females or males (ANOVA, each *p* < 0.05), indicating the specific selectivity distribution of these pollutants in frog tissues. Overall, the liver tissue with blood-rich perfusion and active metabolism showed significantly higher pollutant concentrations in these two frog species, whether for females or males. Relatively high concentrations of PFRs were also observed in the livers of some wild freshwater fish species (i.e., mud carp, snakehead, crucian carp and loach) and marine fish [20,21,26,32], which were consistent with our finding. Wu et al. [35] and Kim et al. [36] found that the liver preferentially accumulates polybrominated diphenyl ethers (PBDEs) in wild rice frogs and seven freshwater fish species, which benefited from the active accumulation and lipid enrichment of the liver. Our previous study also found a strong correlation between PFR concentrations and the lipid content of tissues (i.e., liver, kidney, gill, muscle) in snakehead and mud carp [21]. Meanwhile, the rapid metabolism and biotransformation of PFRs and plasticizers in liver tissue were also important factors, which caused the relatively low accumulation in other tissues [20,30]. Noteworthy, compared with most of other tissues (i.e., lung, muscle, and heart), the egg/gonad tissues also exhibited generally higher PFR concentrations, and the gonads showed higher plasticizer levels in these two frog species, indicating a high risk of parental transfer on these contaminants for the offspring. The relatively high contamination of egg/gonad tissues is attributed to the efficient parental transfer of pollutants [24].

As for the composition patterns of these pollutants, PFRs were dominated by tris(2-chloroethyl) phosphate (TCEP) (12–42%) and tris(chloro-2-propyl) phosphate (TCIPP) (13–47%) in most of black-spotted frog tissues. The exceptions were for the female tissues of kidney, heart, and intestine, tris(2-butoxyethyl) phosphate (TBOEP) (26%) and triphenyl phosphate (TPHP) (24%) were dominant in the kidney, and TCEP (24% and 25%, respectively) and TPHP (20% and 18%) were dominant in the heart and intestine. In all bullfrog tissues, TCEP (23–72%) was the predominant PFR pollutants. As for plasticizers, it was commonly dominated by di-2-ethylhexyl-phthalate (DEHP) (7.0–78%) and di-*n*-butyl-phthalate (DnBP) (3.0–75%), followed by di-*iso*-butyl-phthalate (DiBP) (1.0–34%) in tissue samples of these two frog species.

### 3.2. Tissue-Specific Distribution of PFRs and Plasticizers in Frogs

**Figure 2:**
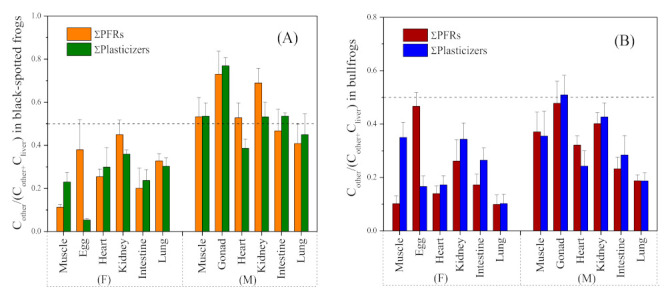
OLR values in (**A**) black-spotted frogs and (**B**) bullfrogs. Error bars represent standard errors. F and M represent female and male, respectively. Data on the tissues of muscle and egg/gonad taken from our previous study [24].

To further examine the distribution of PFRs and plasticizers among these seven different tissues in frogs, the ratios of pollutant concentrations in other tissues to sum (livers + other tissues) (OLR, *C*_other_/(*C*_other_ + *C*_liver_)) were calculated. When OLR was significantly deviated from 0.5, it indicated the significant difference in distribution of PFRs and plasticizers between other tissues and the liver [21,37]. The calculated OLR values of total PFRs and plasticizers for six tissues were 0.112–0.450 and 0.054–0.360 in female black-spotted frogs, 0.408–0.730 and 0.386–0.770 in male black-spotted frogs, 0.099–0.467 and 0.102–0.350 in female bullfrogs, and 0.187–0.478 and 0.187–0.509 in male bullfrogs, respectively. Most OLR values were significantly less than 0.5 (Figure 2), again indicating the significantly higher pollutant concentrations in liver tissues than the others or the selectivity of tissue distribution for PFRs and plasticizers in frogs. These calculated OLR values of PFRs were commonly lower than those in fish tissues (i.e., muscle and kidney) [21,26], suggesting that compared with fish, the frog liver might have a higher accumulation potential or a relatively low metabolic potential for PFRs. In addition, the OLR values of male frogs were generally significantly greater than those of females, implying that the male frogs had higher metabolic capacities on PFRs and plasticizers than the females (Figure 2), which is in line with our previous results [24]. 

The correlation analysis (Table S4) showed that there were significant correlations on the ∑PFRs and ∑plasticizers between livers and intestines, and between kidneys and lungs in black-spotted frogs and bullfrogs. Meanwhile, significant correlations on the ∑plasticizers between livers and kidneys, between livers and lungs, and between hearts and lungs were also observed. These strong correlations between lungs/intestines and other tissues with relatively large blood perfusion (i.e., liver, kidney) might be related to the release of pollutants through breathing and excretion. It is worth noting that the PFR concentrations in livers are significantly and positively related with those in eggs, but no correlation between livers and gonads was found. As Crawshaw and Weinkle [38] suggested, the liver tissue is the organ for the production of egg yolk in female frogs, which could be responsible for the positive correlation between livers and eggs in this study.

Considering these ratios varied among different chemicals, the relationships between OLR ratios corresponding to each frog tissues and log *K*_OW_ of PFRs and plasticizers were further investigated. Exceptions existed for the intestines in male black-spotted frogs, and lungs in male bullfrogs (Figure S2), where no significant correlations were observed, indicating that the lipophicity may have little effect on the deposition of PFRs and plasticizers in frog livers. Since PFRs and plasticizers are easily metabolized in organisms [23], these results could be affected by the bioaccumulation pathway and metabolism.

### 3.3. Relationships between Physiological Parameters and Pollutant Concentrations in Frog Livers 

The hepatosomatic index (HSI) calculated as the ratio of liver weight to body weight, has been conveniently used for estimating the energy status [39] and contaminant exposure as biomarkers [40]. In this study, the relationships between HSI and contaminant concentrations in liver tissues were tentatively examined for bullfrogs since the bullfrog tissue samples were individually analyzed. Strong and negative correlations between HSI and ∑PFRs, ∑plasticizers were observed in female bullfrogs (Figure S3, r = −0.804 and −0.704, each *p* > 0.05), suggesting that the high exposure of PFRs and plasticizers could tend to shrink the livers of these frogs [41]. Du et al. [41] also found the HSI was significantly and negatively correlated with the CP levels in frog livers, which is in agreement with our finding. Schwaiger et al. [42] and Zaroogian et al. [43] pointed out that the reduced livers of carp and flounder after estrogen exposure feeding may be the result of the reduction of liver glycogen deposits, considering that the elimination of pollutants requires energy, which was provided by the consumption of glycogen [41]. 

Since the body weight and snout-vent lengths (SVL) are often used to represent the physical condition of creature, the relationships between body weight (or SVL) and contaminant concentrations in frog livers, and between body weight (or SVL) and HSI were further investigated. Significantly negative correlation was observed between the total PFR concentrations and SVL in female bullfrogs (Figure S3, r = −0.804 and *p* = 0.009). Additionally, significantly positive correlations were found between HSI and SVL, and between HSI and body weight in male bullfrogs (Figure S3, r = 0.835 and 0.945, *p* = 0.039 and 0.008). These findings could indicate that the high exposure to PFRs and plasticizers may reduce the energy storage in frog livers, and further reduce the survival rate of frogs during hibernation [41]. However, more data are needed to reveal the ecological risks of high exposure of PFRs and plasticizers to frogs due to the small sample size of this study.

### 3.4. Parental Transfer Patterns in Frogs Accessed Using Different Tissues as Parental Tissues

The parental transfer characteristics of PFRs and plasticizers in these frogs were investigated by using muscle tissues as parental tissues in our recent study [24]. In a recent laboratory exposure experiment using hen as a model organism, Li et al. [44] found different maternal transfer patterns of halogenated organic contaminants (e.g., PBDEs, polychlorinated biphenyls, dechlorane plus) when using different tissues as maternal tissues, and suggested that the liver, fat, kidney, and the intestine could be selected as more suitable tissues for evaluating maternal transfer of these chemicals. As a tentative investigation, parental transfer ratios (EMR, eggs/maternal tissues in the females; GMR, gonads/paternal tissues in the males) of PFRs and plasticizers were also calculated by using other tissues, including livers, kidneys, hearts, intestines, and lungs, as parental tissues in black-spotted frogs and bullfrogs in this study. 

**Figure 3:**
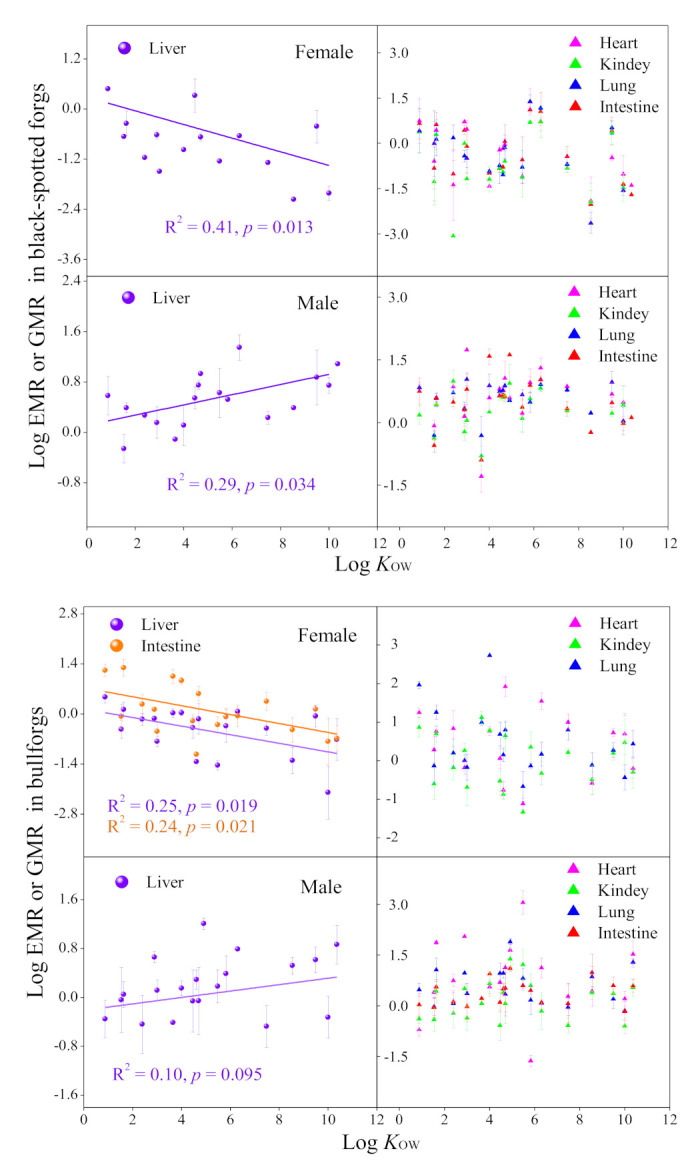
Relationships between the parental transfer ratios accessed by using different tissues as parental tissues with log *K*_OW_ of PFRs and plasticizers. The bars represent standard errors.

In these two female frog species, when the livers were used as maternal tissues, significantly negative linear correlations between log EMR and log *K*_OW_ were observed (Figure 3), which is in accordance with the previous results assessed by using the muscles as maternal tissues [24]. The liver tissue is the organ for the production of yolk proteins [38], which was commonly used as the representative tissue in frogs when evaluating the maternal transfer of some hydrophobic halogenated organic pollutants (e.g., PBDEs, chlorinated paraffin) [35,41]. Additionally, the intestine tissue of female bullfrog also showed the same correlation. For male frogs, significantly positive correlations were found between log GMR and log *K*_OW_ when using liver tissues as paternal tissues (Figure 3). The log GMR significantly increased with log *K*_OW_ when log *K*_OW_ < 6, and then decreased, when using the muscles as paternal tissues in frogs [24]. No obvious correlations were found when the hearts, kidneys, and lungs were used for evaluation (Figure 3). Therefore, when using different tissues as parental tissues, the parental transfer patterns for PFRs and plasticizers in frogs seemed to be different. Moreover, the liver tissues had similar parental transfer mechanisms with muscles. However, more investigations are needed to reveal and clarify it.

## 4. Conclusions

In this study, the internal exposure of PFRs and plasticizers in wild amphibian frog tissues were investigated. Overall, livers exhibited significantly higher contaminant concentrations among different tissues in black-spotted frogs and bullfrogs, as evidenced by the fact that most OLR values were significantly less than 0.5. The OLR values corresponding to the paired tissues in male frogs were significantly greater than those in females, indicating that male frogs could have higher metabolic capacities of PFRs and plasticizers. The lack of significance between OLR ratios and log *K*_OW_ suggested that the other factors (e.g., bioaccumulation pathway and metabolism) besides lipophicity could influence the deposition of PFRs and plasticizers in frog livers. The high exposure to PFRs and plasticizers may reduce the energy storage in frog liver, and further reduce the survival rate of frogs during hibernation. Additionally, different parental transfer patterns for PFRs and plasticizers assessed by using different tissues as parental tissues were found. Moreover, the liver exhibited similar mechanisms with the muscle in frogs. Due to the high metabolic potential of PFRs and plasticizers, more investigations on the metabolites are recommended to comprehensively understand the mechanism and kinetics of the tissue-specific accumulation of PFRs and plasticizers in amphibians.

## Figures and Tables

**Table 1:** Concentrations (average ± standard deviation, ng/g ww) of PFRs and plasticizers in black-spotted frog tissues.

Tissues	Female Black-Spotted Frogs					Male Black-Spotted Frogs				
	Liver	Heart	Kidney	Intestine	Lung	Liver	Heart	Kidney	Intestine	Lung
*N*	3	3	3	3	3	3	3	3	3	3
**TEP**	0.18 ± 0.17	0.070 ± 0.030	0.16 ± 0.070	0.08 ± 0.040	0.14 ± 0.060	0.040 ± 0.010	0.020 ± 0.010	0.10 ± 0.010	0.030 ± 0.010	0.030 ± 0.020
**TCEP**	2.8 ± 0.55	1.0 ± 0.44	1.4 ± 0.73	0.64 ± 0.25	2.1 ± 1.1	0.89 ± 0.25	0.82 ± 0.18	0.81 ± 0.32	0.58 ± 0.19	0.56 ± 0.20
**TCIPP**	6.0 ± 0.50	0.030 ± 0.050	0.67 ± 0.38	0.30 ± 0.44	1.6 ± 0.83	0.76 ± 0.78	0.72 ± 0.83	1.3 ± 1.0	0.32 ± 0.070	0.29 ± 0.030
**TNBP**	0.68 ± 0.32	0.40 ± 0.33	0.56 ± 0.73	0.16 ± 0.11	0.28 ± 0.15	0.30 ± 0.12	0.070 ± 0.070	0.040 ± 0.070	0.020 ± 0.020	0.040 ± 0.030
**TDCIPP**	1.0 ± 0.99	0.61 ± 0.38	1.0 ± 0.82	0.22 ± 0.090	0.62 ± 0.59	0.010 ± 0.020	0.31 ± 0.16	0.10 ± 0.11	0.040 ± 0.030	0.10 ± 0.090
**TPHP**	0.62 ± 0.45	0.90 ± 0.75	3.4 ± 3.9	0.62 ± 0.54	0.84 ± 0.43	0.14 ± 0.13	0.23 ± 0.17	0.33 ± 0.060	0.27 ± 0.11	0.12 ± 0.12
**TBOEP**	0.29 ± 0.41	0.050 ± 0.080	2.8 ± 1.7	1.2 ± 2.0	0.42 ± 0.66	ND	0.020 ± 0.030	1.5 ± 1.2	0.20 ± 0.15	0.050 ± 0.050
**EHDPHP**	0.99 ± 1.0	0.35 ± 0.39	0.22 ± 0.12	0.13 ± 0.11	0.060 ± 0.10	0.14 ± 0.13	0.090 ± 0.070	0.36 ± 0.24	0.14 ± 0.060	0.12 ± 0.11
**TpTP**	0.050 ± 0.010	0.28 ± 0.29	0.18 ± 0.10	0.060 ± 0.060	0.090 ± 0.070	0.020 ± 0.020	0.060 ± 0.060	0.070 ± 0.030	0.030 ± 0.010	0.020 ± 0.010
**TEHP**	0.15 ± 0.070	0.33 ± 0.31	0.080 ± 0.070	0.070 ± 0.060	0.060 ± 0.060	0.070 ± 0.080	0.080 ± 0.040	0.19 ± 0.040	0.10 ± 0.010	0.060 ± 0.050
**iDDPHP**	0.14 ± 0.040	0.31 ± 0.25	0.46 ± 0.13	0.18 ± 0.18	0.22 ± 0.11	0.050 ± 0.070	0.070 ± 0.030	0.12 ± 0.050	0.040 ± 0.020	0.050 ± 0.050
**RDP**	ND	0.010 ± 0.010	0.020 ± 0.020	0.010 ± 0.010	ND	ND	ND	ND	ND	0.010 ± 0.010
**BDP**	0.15 ± 0.040	0.18 ± 0.20	0.44 ± 0.36	0.12 ± 0.10	0.070 ± 0.060	0.040 ± 0.060	0.010 ± 0.010	0.040 ± 0.020	0.090 ± 0.15	0.010 ± 0.010
**∑PFRs**	13 ± 4.6	4.50 ± 3.5	11 ± 9.1	3.8 ± 4.0	6.5 ± 4.2	2.5 ± 1.7	2.5 ± 1.7	5.0 ± 3.1	1.9 ± 0.82	1.4 ± 0.78
**DMP**	2.3 ± 2.5	20 ± 33	83 ± 91	25 ± 19	16 ± 24	52 ± 30	33 ± 18	61± 20	105 ± 82	55 ± 29
**DEP**	5.7 ± 3.0	57 ± 82	90 ± 156	39 ± 21	6.8 ± 8.3	60 ± 55	31 ± 11	14 ± 13	77 ± 44	52 ± 45
**DiBP**	44 ± 33	49 ± 29	354 ± 472	182 ± 77	238 ± 213	278 ± 180	139 ± 51	202 ± 59	196 ± 47	164 ± 73
**DnBP**	2066 ± 1816	858 ± 1190	691 ± 378	481 ± 226	842 ± 79	514 ± 165	477± 88	717 ± 125	626 ± 137	499 ± 181
**BBzP**	1.2 ± 0.77	29 ± 49	0.62 ± 1.1	2.5 ± 4.4	1.1 ± 1.5	2.2 ± 3.9	3.9 ± 3.5	1.3 ± 1.6	0.15 ± 0.26	0.74 ± 1.3
**DEHP**	3081 ± 220	1443 ± 1311	1773 ± 1186	884 ± 724	1142 ± 400	551 ± 362	160 ± 11	503 ± 229	515 ± 366	251 ± 100
**DEHT**	45 ± 24	10 ± 8.0	36 ± 49	14 ± 6.0	26 ± 17	4.6 ± 4.2	12 ± 12	12 ± 9.5	24 ± 13	26 ± 28
**DIDP**	16 ± 2.5	28 ± 24	64 ± 20	84 ± 48	88 ± 104	4.8 ± 7.0	ND	ND	40 ± 17	30 ± 22
**DINCH**	229 ± 190	86 ± 112	ND	58 ± 42	82 ± 117	7.2 ± 7.7	ND	45 ± 79	81 ± 61	40 ± 38
**∑Plasticizers**	5503 ± 2181	2584 ± 1601	3103 ± 444	1772 ± 846	2448 ± 822	1478 ± 739	858 ± 116	1561 ± 38	1666 ± 701	1120 ± 317

*N*, the number of composite samples; ND, not detected; TEP, triethyl phosphate; TCEP, tris(2-chloroethyl) phosphate; TCIPP, tris(chloro−2-propyl) phosphate; TNBP, tri-*n*-butyl phosphate; TDCIPP, tris(1,3-dichloro-2-propyl) phosphate; TPHP, triphenyl phosphate; TBOEP, tris(2-butoxyethyl) phosphate; EHDPHP, 2-ethylhexyl diphenyl phosphate; TpTP, tri-cresyl phosphate; TEHP, tris(2-ethylhexyl) phosphate; iDDPHP, *iso*-decyl diphenyl phosphate; RDP, resorcinol bis(diphenylphosphate); BDP, bisphenol A-bis (diphenyl phosphate); DMP, dimethyl-phthalate; DEP, diethyl-phthalate; DiBP, di-*iso*-butyl-phthalate; DnBP, di-*n*-butyl-phthalate; BBzP, benzyl-butyl-phthalate; DEHP, di-2-ethylhexyl-phthalate; DEHT, bis-(2-ethylhexyl) terephthalate; DIDP, di-*iso*-decyl phthalate; DINCH, di-*iso*-nonylcyclohexane-1,2-dicarboxylate.

**Table 2:** Concentrations (average ± standard deviation, ng/g ww) of PFRs and plasticizers in bullfrog tissues.

Tissues	Female Bullfrogs					Male Bullfrogs				
	Liver	Heart	Kidney	Intestine	Lung	Liver	Heart	Kidney	Intestine	Lung
*N*	5	5	5	5	5	5	5	5	5	5
**TEP**	1.9 ± 1.8	0.070 ± 0.10	0.55 ± 0.67	0.30 ± 0.23	0.060 ± 0.050	0.70 ± 0.45	0.14 ± 0.20	0.53 ± 0.40	0.18 ± 0.15	0.040 ± 0.050
**TCEP**	19 ± 16	4.0 ± 2.1	6.7 ± 7.2	1.6 ± 1.7	1.5 ± 1.1	9.5 ± 6.6	5.3 ± 2.3	4.1 ± 5.1	1.8 ± 1.7	1.1 ± 1.0
**TCIPP**	0.62 ± 0.22	0.77 ± 0.40	0.25 ± 0.21	0.16 ± 0.15	0.16 ± 0.23	0.48 ± 0.17	0.83 ± 0.25	0.25 ± 0.28	ND	0.030 ± 0.060
**TNBP**	0.56 ± 0.20	0.11 ± 0.050	0.10 ± 0.030	0.070 ± 0.030	ND	0.31 ± 0.21	0.090 ± 0.030	0.10 ± 0.060	0.010 ± 0.030	ND
**TDCIPP**	0.59 ± 0.33	ND	0.030 ± 0.030	0.070 ± 0.040	0.050 ± 0.070	0.26 ± 0.20	ND	0.080 ± 0.070	0.050 ± 0.060	0.020 ± 0.030
**TPHP**	17 ± 25	0.010 ± 0.020	1.3 ± 1.2	1.2 ± 1.1	0.56 ± 0.39	1.9 ± 1.4	0.11 ± 0.12	0.79 ± 0.35	0.49 ± 0.42	0.47 ± 0.30
**TBOEP**	2.0 ± 1.6	ND	2.1 ± 1.7	0.75 ± 0.49	0.49 ± 0.49	0.52 ± 0.46	0.030 ± 0.040	1.8 ± 2.2	1.7 ± 3.0	0.89 ± 1.6
**EHDPHP**	0.49 ± 0.55	0.020 ± 0.030	0.86 ± 0.72	0.69 ± 0.33	0.23 ± 0.31	0.29 ± 0.35	0.010 ± 0.030	1.9 ± 1.9	0.27 ± 0.23	0.17 ± 0.24
**TpTP**	1.1 ± 0.43	0.56 ± 0.22	0.57 ± 0.65	0.090 ± 0.030	0.26 ± 0.21	0.84 ± 0.60	0.63 ± 0.15	0.080 ± 0.10	0.11 ± 0.070	0.22 ± 0.28
**TEHP**	0.54 ± 0.45	0.050 ± 0.040	0.16 ± 0.080	0.17 ± 0.040	0.16 ± 0.11	0.23 ± 0.17	0.050 ± 0.060	0.26 ± 0.21	0.18 ± 0.16	0.33 ± 0.23
**iDDPHP**	0.26 ± 0.45	ND	0.040 ± 0.050	0.10 ± 0.090	0.080 ± 0.12	0.080 ± 0.080	ND	0.080 ± 0.080	0.020 ± 0.030	0.040 ± 0.040
**RDP**	0.49 ± 0.46	ND	0.070 ± 0.060	0.13 ± 0.080	0.030 ± 0.040	0.17 ± 0.16	ND	0.18 ± 0.25	0.020 ± 0.040	0.12 ± 0.24
**BDP**	0.76 ± 0.77	0.32 ± 0.70	0.13 ± 0.070	2.3 ± 4.5	0.060 ± 0.060	0.19 ± 0.19	ND	0.37 ± 0.66	0.050 ± 0.060	0.18 ± 0.23
**∑PFRs**	46 ± 29	5.9 ± 2.5	13 ± 6.8	7.6 ± 4.1	3.6 ± 1.2	16 ± 4.4	7.2 ± 2.2	10 ± 3.7	4.9 ± 2.8	3.6 ± 1.3
**DMP**	23 ± 12	5.5 ± 4.5	45 ± 34	9.7 ± 4.5	14 ± 12	39 ± 45	5.5 ± 5.5	32 ± 24	7.55 ± 6.2	13 ± 9.6
**DEP**	29 ± 28	4.9 ± 4.7	36 ± 28	9.1 ± 3.8	13 ± 11	48.33 ± 57.39	3.1 ± 3.2	29 ± 26	5.36 ± 3.6	12 ± 7.8
**DiBP**	252 ± 132	77 ± 33	731 ± 624	120 ± 54	34 ± 28	435 ± 748	53 ± 53	485 ± 560	97 ± 73	29 ± 25
**DnBP**	1146 ± 751	514 ± 223	609 ± 161	861 ± 459	174 ± 148	766 ± 821	430 ± 264	412 ± 297	412 ± 339	155 ± 117
**BBzP**	8.0 ± 8.9	0.42 ± 0.57	0.51 ± 0.74	1.1 ± 1.0	0.11 ± 0.14	0.85 ± 0.84	0.18 ± 0.34	0.37 ± 0.59	0.13 ± 0.14	0.060 ± 0.020
**DEHP**	1542 ± 1430	74 ± 96	286 ± 102	321 ± 225	96 ± 101	969 ± 859	146 ± 165	707 ± 352	378 ± 415	217 ± 128
**DEHT**	64 ± 48	72 ± 158	18 ± 8.3	25 ± 20	9.6 ± 8.0	30 ± 32	20 ± 23	29 ± 22	20 ± 16	13 ± 18
**DIDP**	128 ± 109	4.3 ± 5.9	56 ± 45	65 ± 28	9.8 ± 5.9	49 ± 48	11 ± 18	53 ± 40	31 ± 26	7.9 ± 5.8
**DINCH**	312 ± 376	0.54 ± 1.2	5.2 ± 5.6	11 ± 18	3.0 ± 2.4	6.3 ± 7.6	3.2 ± 2.9	10 ± 8.3	11 ± 7.4	18 ± 34
**∑Plasticizers**	3504 ± 1067	753 ± 487	1787 ± 645	1423 ± 712	355 ± 184	2344 ± 1399	672 ± 487	1758 ± 992	961 ± 819	465 ± 148

*N*, the number of samples; ND, not detected.

## Data Availability

Data is available from the corresponding author by request.

## Supplementary Materials

The following are available online at https://www.mdpi.com/article/10.3390/toxics9060124/s1, Section S1: Chemicals and Instrument Analysis, Figure S1: Total concentrations of PFRs and plasticizers in each tissue of black-spotted frogs and bullfrogs, Figure S2: Relationships between the OLR ratios in frogs and log *K*_OW_ of PFRs and plasticizers, Figure S3: Relationships between physiological parameters and PFRs and plasticizers burdens in bullfrog livers, Table S1: Overview for the targeted PFR and plasticizer chemicals in this study, Table S2: The procedure blank contamination levels of each chemical (detected units in instrument: ng/mL), and the average limit of quantification of each chemical in analyzed samples (ng/g ww), Table S3: Recoveries (mean ± SD) of seven surrogate standards in the present samples, Table S4: Correlations on total PFR and plasticizer concentrations among different tissues in frogs.
