# Supplementary material for: Tissue-Specific Distribution of Legacy and Emerging Organophosphorus Flame Retardants and Plasticizers in Frogs
Source: Toxics. 2021 May 31;9(6):124. doi: 10.3390/toxics9060124 (PMC8228168; doi:10.3390/toxics9060124)
Supplement: Supplementary file 1 [file toxics-09-00124-s001.zip › toxics-1221616-SI.pdf]

toxics

Supplementary materials: Tissue-specific Distribution of Legacy and Emerging Organophosphorus Flame Retardants and Plasticizers in Frogs

Yin-E Liu, Xiao-Jun Luo, Ke-Lan Guan, Chen-Chen Huang, Xue-Meng Qi, Yan-Hong Zeng and Bi-Xian Mai

Chemicals and Instrument Analysis

Thirteen PFR chemicals (including 10 legacy PFRs and 3 emerging PFRs) and nine plasticizers (including 6 legacy phthalates and 3 alternative plasticizers) were analyzed in the present study. Labeled tri-*n*-butyl phosphate (TNBP-d27), tris(2-chloroethyl) phosphate (TCEP-d12), tris(1,3-dichloro-2-propyl) phosphate (TDCIPP-d15), and triphenyl phosphate (TPHP-d15) were used as surrogate standards (IS) for the quantitative analyses of PFRs, and di-*n*-butyl-phthalate (DNBP-d4), dibenzyl phthalate (DBzP-d4), and di-2-ethylhexyl-phthalate (DEHP-d4) were used for the quantitative analyses of plasticizers. Triamyl phosphate (TAP) was used as recovery standards.

Identification of PFRs and plasticizers was performed by an Agilent Triple Quadrupole LC-MS/MS with an electrospray ionization (ESI) source and a Phenomenex Kinetex Biphenyl reversed phase column (2.1 × 100 mm, 2.6 μm; Torrance, CA, USA). The column temperature was set as 40 °C. The mobile phases were A) UPW with 5 mM ammonium formate, and B) methanol with 5 mM ammonium formate. The gradient of separation was: from 30% (B) increased to 70% (B) in 5 mins, reached 100% at 20 min, and hold for 5 mins, finally, returned to initial conditions (30% (B)) and kept equilibration for 10 mins. The total duration of each injection was 35 min and with an injection flow of 0.25 mL/min and injection volume of 5 μL. The dynamic multiple reaction monitoring (dMRM) was operated for mass spectrometer in positive ionization mode (time segments were set at 1.5 min for each compound). MRM transitions of target analytes were presented in the previous study [1].

DEHP and DEHT were detected with a Shimadzu GC/MS operated in electron ionization mode (EI). The analytes separation was performed with a DB-5 column (30 m × 0.25 mm, 0.25 μm). The injection temperature was set at 80 °C, and ramped at 40 °C/min to 280 °C, held 10 min, then ramped at 80 °C/min to 300 °C, held 15 min. The ion source temperature was set as 250 °C, and injection volume was 1 μL. Helium was used as a carrier gas with a flow rate of 0.91 mL/min [2].

Table S1. Overview [2] for the targeted PFR and plasticizer chemicals in this study.

	Compound Name	Acronym	Molecular Mass (g/mol)	Log *K*ow
PFRs	Triethyl phosphate	**TEP**	182.15	0.87
	Tris(2-chloroethyl) phosphate	**TCEP**	285.49	1.63
	Tris(chloro-2-propyl) phosphate	**TCIPP**	327.57	2.89
	Tri-*n*-butyl phosphate	**TNBP**	266.31	4
	Tris(1,3-dichloro-2-propyl) phosphate	**TDCIPP**	430.90	3.65
	Triphenyl phosphate	**TPHP**	326.28	4.7
	Tris(2-butoxyethyl) phosphate	**TBOEP**	398.47	3
	2-ethylhexyl diphenyl phosphate	**EHDPHP**	362.39	6.3
	Tri-cresyl phosphate	**TPTP**	368.36	5.48
ePFRs	Tris(2-ethylhexyl) phosphate	**TEHP**	434.63	9.49
	Isodecyl diphenyl phosphate	**iDDPHP**	390.45	5.44
	Resorcinol bis(diphenylphosphate)	**RDP**	574.45	5.82
	Bisphenol A- bis (diphenyl phosphate)	**BDP**	692.63	4.5
LPs	Dimethyl-phthalate	**DMP**	194.18	1.53
	Diethyl-phthalate	**DEP**	222.24	2.39
	Di-*iso*-butyl-phthalate	**DiBP**	278.34	4.46
	Di-*n*-butyl-phthalate	**DnBP**	278.34	4.61
	Benzyl-butyl-phthalate	**BBzP**	312.36	4.91
APs	Di-2-ethylhexyl-phthalate	**DEHP**	390.56	7.48
	Bis-(2-ethylhexyl) terephthalate	**DEHT**	390.56	8.54
	Di-*iso*-decyl phthalate	**DIDP**	446.67	10.36
	Di-iso-nonylcyclohexane-1,2-dicarboxylate	**DINCH**	424.66	10

The data are taken from studies [3-6].

Table S2. The procedure blank contamination levels of each chemical (detected units in instrument: ng/mL), and the average limit of quantification of each chemical in analyzed samples (ng/g ww).

Chemicals	Blank (ng/mL)	SD	Liver	Kidney	Intestine	Lung	Heart
**TEP**	1.2	0.19	0.34	0.44	0.32	0.38	0.50
**TCEP**	3.3	0.46	0.87	1.1	0.82	0.97	1.3
**TCIPP**	6.3	0.47	1.4	1.8	1.3	1.6	2.1
**TNBP**	2.1	0.23	0.50	0.64	0.47	0.55	0.72
**TDCIPP**	2.9	0.61	0.85	1.1	0.79	0.95	1.2
**TPHP**	0.88	0.12	0.25	0.32	0.23	0.27	0.36
**TBOEP**	0.31	0.038	0.079	0.10	0.074	0.087	0.11
**EHDPHP**	1.2	0.069	0.27	0.34	0.25	0.30	0.39
**TPTP**	0.41	0.0089	0.077	0.098	0.072	0.085	0.11
**TEHP**	0.41	0.0095	0.080	0.10	0.075	0.089	0.12
**iDDPHP**	0.49	0.041	0.12	0.15	0.11	0.13	0.17
**RDP**	0.25	0.022	0.059	0.076	0.055	0.066	0.086
**BDP**	0.34	0.048	0.089	0.11	0.083	0.099	0.13
**DMP**	17	3.2	9.1	12	8.5	10	13
**DEP**	29	5.3	15	19	14	16.	22
**DiBP**	62	20	23	30	22	26	34
**DnBP**	102	31	36	46	34	40	52
**BBzP**	4.7	0.43	1.2	1.5	1.1	1.3	1.7
**DEHP**	434	121	147	188	137	163	213
**DEHT**	98	2.3	28	35	26	31	40
**DINCH**	1.9	0.37	0.55	0.71	0.51	0.61	0.80
**DIDP**	11	0.91	2.6	3.4	2.4	2.9	3.8

SD: standard deviation.

Table S3. Recoveries (mean ± SD) of seven surrogate standards in the present samples.

**TNBP-d27**	87 ± 12
**TPHP-d15**	84 ± 14
**TDCIPP-d15**	89 ± 10
**TCEP-d12**	90 ± 21
**DnBP-d4**	83 ± 6.3
**DBzP-d4**	93 ± 16
**DEHP-d4**	82 ± 12

Table S4. Correlations on total PFR and plasticizer concentrations among different tissues in frogs.

Total PFRs	Muscle	Egg/gonad	Liver	Heart	Kidney	Intestine	Lung
Muscle	1						
Egg/gonad	0.168	1					
Liver	−0.035	0.842**	1				
Heart	0.263	0.408	0.341	1			
Kidney	0.044	0.319	0.419	0.144	1		
Intestine	−0.203	0.316	0.655**	0.321	0.246	1	
Lung	−0.214	0	0.204	0.256	0.601**	0.096	1

Total plasticizers	Muscle	Egg/gonad	Liver	Heart	Kidney	Intestine	Lung
Muscle	1						
Egg/gonad	−0.104	1					
Liver	0.145	−0.578*	1				
Heart	−0.062	−0.263	0.151	1			
Kidney	0.011	−0.375	0.674**	0.318	1		
Intestine	−0.144	0.060	0.459*	0.329	0.197	1	
Lung	0.052	−0.136	0.467*	0.449*	0.601**	0.177	1

* $p < 0.05$, ** $p < 0.01$.

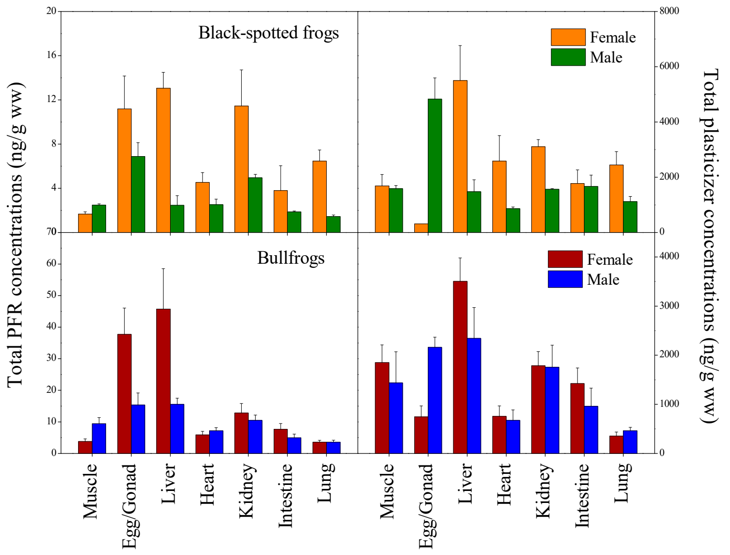

Figure S1. Total concentrations of PFRs and plasticizers in each tissue of black-spotted frogs and bullfrogs. Error bars represent standard errors.

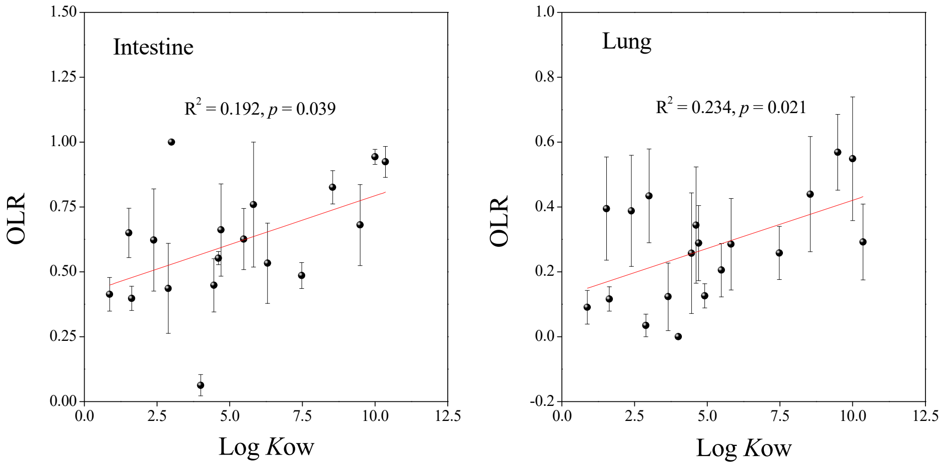

Figure S2. Relationships between the OLR ratios in frogs and log *K*ow of PFRs and plasticizers.

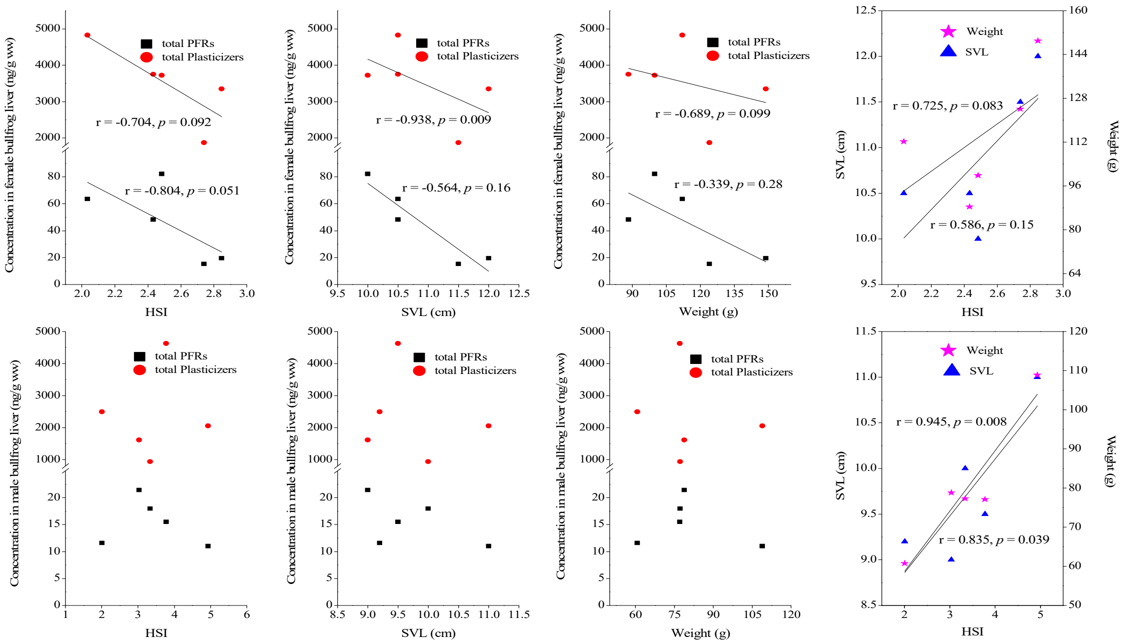

Figure S3. Relationships between physiological parameters and PFRs and plasticizers burdens in bullfrog livers.

References

1. Christia, C.; Tang, B.; Yin, S.S.; Luo, X.J.; Mai, B.X.; Poma, G.; Covaci, A. Simultaneous determination of legacy and emerging organophosphorus flame retardants and plasticizers in indoor dust using liquid and gas chromatography–tandem mass spectrometry: method development, validation, and application. *Anal. Bioanal. Chem.* **2019**, *411*, 7015-7025.
2. Liu, Y.E; Luo, X.J.; Guan, K.L.; Huang, C.C.; Zhu, C.Y.; Qi, X.M.; Zeng, Y.H.; Mai, B.X. Legacy and emerging organophosphorus flame retardants and plasticizers in frogs: Sex difference and parental transfer. *Environ. Pollut*. **2020**, *266*, 115336.
3. Bui, T.T.; Giovanoulis, G.; Cousins, A.P.; Magner, J.; Cousins, I.T.; de Wit, C.A. Human exposure, hazard and risk of alternative plasticizers to phthalate esters. *Sci. Total Environ.* **2016**, 541, 451-467.
4. van der Veen, I.; de Boer, J. Phosphorus flame retardants: Properties, production, environmental occur rence, toxicity and analysis. *Chemosphere* **2012**, *88(10)*, 1119-1153.
5. Hou, R.; Xu, Y.; Wang, Z. Review of OPFRs in animals and humans: Absorption, bioaccumulation, metabolism, and internal exposure research. *Chemosphere* **2016**, *153*, 78-90.
6. Giovanoulis, G.; Bui, T.; Xu, F.; Papadopoulou, E.; Padilla-Sanchez, J. A.; Covaci, A.; Haug, L. S.; Cousins, A. P.; Magner, J.; Cousins,T.; de Wit, C. A. Multi-pathway human exposure assessment of phthalate esters and DINCH. *Environ. Int.* **2018**, *112*, 115-126.
